# Transport Characteristics and Transmission Risk of Virus-Containing Droplets from Coughing in Outdoor Windy Environment

**DOI:** 10.3390/toxics10060294

**Published:** 2022-05-29

**Authors:** Guoyi Jiang, Fengjiao Li, Tingting Hu

**Affiliations:** 1 Department of Civil and Environmental Engineering, Shantou University, Shantou 515063, China; gyjiang@stu.edu.cn; 2 College of Chemistry and Chemical Engineering, Shanghai University of Engineering Science, Shanghai 201620, China; tingtinghu@sues.edu.cn

**Keywords:** cough-jet, COVID-19, droplet dispersion, virtual manikin, computational fluid dynamics, outdoor environment, social distancing, exposure risk

## Abstract

Particle dispersions have been widely studied inside rooms, but few databases have examined the transmission risk of respiratory droplets outdoors. This study investigated the wind effect on the dispersion of coughed droplets and the influence of social distancing on the infection risk in different susceptible persons using computational fluid dynamics simulations. Infection risk was evaluated based on direct depositions and exposure fractions. The results indicated that a reverse and upward flow formed in front of an infected man, and it enhanced as the wind strengthened, which transported more medium particles higher and increased the deposition on both infected and susceptible persons. Small particles moved above the neck, and they rarely deposited on the body. Medium particles larger than 60 μm were more likely to deposit and could reach the head of a healthy person under stronger winds. The exposure fraction achieved peak values when numerous particles passed the breathing zone. Although longer social distancing could alleviate the particle deposition on the face and delay the most dangerous time, its effect on infection risk was ambiguous. The infection risk was larger for a shorter susceptible person because more particles were deposited on the face, and the exposure fraction contributed by particles above the neck was larger.

## 1. Introduction

The epidemic of Coronavirus Disease 2019 (COVID-19), which occurred at the end of the year 2019, has caused an abrupt disruption in the world and resulted in serious destruction to both the economic activity and the lives of normal people. To prevent virus transmission in a local area, lockdowns have been unwillingly adopted sometimes, and societies were cautiously opened up after the alleviation of the disease. COVID-19 is mainly caused by a virus called Severe Acute Respiratory Syndrome Coronavirus 2 (SARS-CoV-2), which is usually contained in the droplets exhaled during respiratory activities, such as sneezing, coughing, talking and even breathing. The virus contained in the respiratory droplets can live for hours and mutate constantly [1], such as the Delta variant, which is much more transmissible. As a result, accurately mastering the respiratory activities is the first step for determining the mechanism of virus transmission in the air. Many experiments have been conducted to test the number and size distributions of exhaled droplets from a respiratory activity [2,3,4,5]. These studies have indicated that the diameters of exhaled droplets usually range from several to thousands of micrometers, and the small and medium particles whose diameters are less than 100 μm account for most of the percentage. To understand the flow dynamics of respiratory activities, both spirometer and particle image velocimetry instruments were utilized to obtain the airflow rate [5,6,7,8]. The above-measured data provided a detailed database and boundary conditions for further study of respiratory droplet motion using a numerical simulation. As airborne transmission is the main route for virus infection, understanding the transport mechanism of respiratory droplets in the air is essential to provide effective guidelines for mitigating a potential disease epidemic. Although theoretical models have been developed and experiments have been conducted to investigate the dispersion of micro-droplets in the air since the outbreak of COVID-19 [9,10,11], most relevant studies were performed by computational fluid dynamics (CFD) simulations because of the flexible settings of scenarios in the CFD model. Because of the strong virus infection risk in enclosed or confined spaces, investigations on particle dispersion in an indoor environment have caused much concern. Such studies included the micro-droplet transport inside airliner cabins [12,13,14,15], in a high-speed rail cabin [16], in a coach bus [17], in hospital wards [10,11], inside elevators [18] and in other ordinary ventilated rooms [19,20,21,22].

In enclosed spaces, ventilation may influence particle dispersion, and a large volume of a room can be filled with germ-containing droplets, which increases the infection risk for people living inside the room. Investigations have shown that although the infection risk is relatively lower, there is still the possibility of infection related to outdoor activities, such as walking, running and cycling [23,24,25]. Studies of virus transmission through outdoor environments are still ongoing. Unlike the situation of indoor spaces, the dispersion of exhaled particles is strongly affected by the meteorological conditions and the flow disturbance caused by a human body in an outdoor area. For large indoor spaces, the scenario is also similar to the outdoor situation if ventilation occurs in a horizontal direction. The investigation of the transport of exhaled droplets in an outdoor environment is important for understanding the transmission risk of both SARS-CoV-2 and other influenza viruses among humans. In order to prevent people from getting infected, social distancing levels of 1, 1.5 and 1.83 m (6 feet) were suggested by different organizations [25,26,27,28]. However, these policies are only for the static airflow condition, and the effectiveness of these distances and the virus transmission risk still need to be examined for windy conditions. Feng et al. [29] investigated the wind speed and air humidity effects on the fate of exhaled droplets in an outdoor environment; they also discussed the effectiveness of the N95 mask that was manufactured without a valve on the potential exposure risks of a healthy human that stood at a social distance of 6 feet away. Li et al. [30] studied the effects of relative humidity on the transport of evaporative droplets in an outdoor space under a wind speed of 2 m/s; the transmission risks were evaluated based on the viral deposition on a person standing 1 or 2 m away from the cougher. Yang et al. [31] investigated the evaporation and transportation of solid-liquid droplets with diameters of 10, 50 and 100 μm in an open outdoor environment; social distancing effects between two people with identical body shapes were discussed under inflow wind speeds of 0.1 and 1.9 m/s.

Although several studies have examined the transport characteristics of respiratory droplets in outdoor spaces, the virtual manikin models used in these studies were not fine enough to accurately reproduce the airflow around a human body. Moreover, the infection risk for a susceptible person was evaluated mainly based on the direct deposition on the body, and the threats from the airborne transmission were not fully assessed. Another limitation of the existing studies lies in the fact that the influence of the body features on the infection risk was not considered. This study aimed to investigate the impact of wind speed and the aerodynamic effects of the flow around a human body on the transport characteristics of coughed droplets outdoors and the influence of social distancing on the infection risk in different susceptible persons under different wind conditions. Near-real manikin models were used in this study to accurately capture the airflow around a human body. The flow and particle dispersion around an isolated human model was first investigated in a long domain to check the effect of wind speed and to investigate the particle dispersion characteristics without any interruption by other barriers located downstream. Then, a male model and a female model with different heights were selected as susceptible persons to study the social distancing influences on the virus transmission risk in different susceptible persons under different wind conditions. The infection risk was evaluated by both direct depositions on a human body and exposure fraction around the nose. The dispersion characteristics of different sizes of particles were analyzed in detail, and the conclusions were finally drawn.

## 2. Turbulence Model Evaluations

The commonly used Reynolds-Averaged Navier-Stokes (RANS) turbulence models in wind engineering and numerical algorithms used in this study were first evaluated to ensure the accuracy of the CFD technique in predicting wind flow around a blunt body. The examined RANS models were a standard *k*–*ε* model, a renormalization group (RNG) *k*–*ε* model and a revised *k*–*ε* model proposed by Kato and Launder [32] (hereafter denoted as the LK *k*–*ε* model).

The governing equations for a standard *k*–*ε* model are continuum, momentum and transport equations for turbulent kinetic energy *k* and turbulent dissipation rate *ε*:

(1) $$\frac{\partial u_{j}}{\partial x_{j}}=0$$

(2) $$\frac{\partial u_{i}}{\partial t}=-\frac{\partial p}{\partial x_{i}}-\frac{\partial \left(u_{i}u_{j}\right)}{\partial x_{j}}+\frac{\partial }{\partial x_{j}}\left\{\left(\nu +v_{t}\right)\left(\frac{\partial u_{i}}{\partial x_{j}}+\frac{\partial u_{j}}{\partial x_{i}}\right)\right\}-\frac{2}{3}\frac{\partial k}{\partial x_{i}}$$

(3) $$\frac{\partial k}{\partial t}=-\frac{\partial \left(ku_{j}\right)}{\partial x_{j}}+\frac{\partial }{\partial x_{j}}\left\{\left(\nu +\frac{v_{t}}{\sigma_{k}}\right)\frac{\partial k}{\partial x_{j}}\right\}+P_{k}-\varepsilon$$

(4) $$\frac{\partial \varepsilon }{\partial t}=-\frac{\partial \left(\varepsilon u_{j}\right)}{\partial x_{j}}+\frac{\partial }{\partial x_{j}}\left\{\left(\nu +\frac{v_{t}}{\sigma_{\varepsilon }}\right)\frac{\partial \varepsilon }{\partial x_{j}}\right\}+\frac{\varepsilon }{k}\left(C_{\varepsilon 1}P_{k}-C_{\varepsilon 2}\varepsilon \right)$$

where *i* = 1, 2, 3 and *j* = 1, 2, 3 indicate the three coordinate directions, *p* is the pressure, *ν* and *ν_t_* are laminar and turbulent viscosity, respectively, and *σ_k_* and *σ_ε_* are model constants.

In an LK *k*–*ε* model, the production term *P_k_* in the turbulent kinetic energy equation is simply modified from the standard *k*–*ε* model and is computed as follows:

(5) $$P_{k}=\nu_{t}S\Omega$$

where *S* and Ω are the magnitudes of the strain rate and vorticity rate, respectively, which are computed as follows:

(6) $$S=\sqrt{\frac{1}{2}{\left(\frac{\partial u_{i}}{\partial x_{j}}+\frac{\partial u_{j}}{\partial x_{i}}\right)}^{2}}$$

(7) $$\Omega =\sqrt{\frac{1}{2}{\left(\frac{\partial u_{i}}{\partial x_{j}}-\frac{\partial u_{j}}{\partial x_{i}}\right)}^{2}}$$

Because the airflow around a human body has some similarity to that around an isolated high-rise building, wind tunnel experiments of airflow around a building with a 1:1:2 shape [33] were used to validate the current CFD models. The experiment was conducted in a wind tunnel at the Tokyo Polytechnic University. A high-rise building model with a height of 0.2 m and a width of 0.1 m was located in a fully developed turbulent boundary layer. The wind flow around the building was measured using a split film probe. The Reynolds number based on the building width and inflow velocity at building height *U_h_* was about 28,000. The calculated wind velocities using the current turbulence models were compared with the measured flow data.

**Figure 1:**
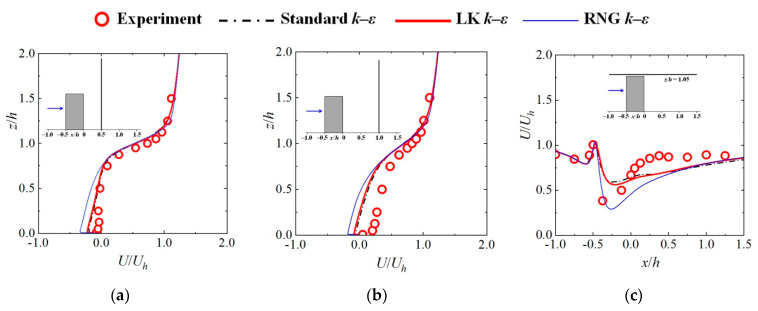

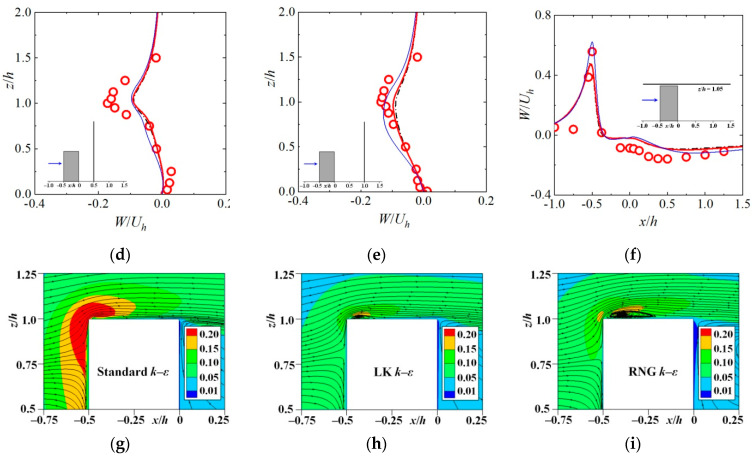
Comparisons between the CFD results with the wind tunnel experiments: (**a**) *U*/*U_h_* (*x*/*h* = 0.5); (**b**) *U*/*U_h_* (*x*/*h* = 1.0); (**c**) *U*/*U_h_* (*z*/*h* = 1.05); (**d**) *W*/*U_h_* (*x*/*h* = 0.5); (**e**) *W*/*U_h_* (*x*/*h* = 1.0); (**f**) *W*/*U_h_* (*z*/*h* = 1.05); (**g**) *k*/*U_h_*^2^ (standard *k*–*ε*); (**h**) *k*/*U_h_*^2^ (LK *k*–*ε*); (**i**) *k*/*U_h_*^2^ (RNG *k*–*ε*).

The normalized streamwise and vertical velocities are compared in Figure 1a–c and 1d–f, respectively. The compared positions included two vertical lines in the wake behind the building (*x*/*h* = 0.5, *x*/*h* = 1.0) and a horizontal line above the building (*z*/*h* = 1.05). The CFD results obtained using the three turbulence models were in accordance with the experimental measurements except in small areas. The difference between the results of the standard and LK *k*–*ε* models was small. In the wake region behind the building, the prediction accuracies of the standard and LK *k*–*ε* models were better than that of the RNG model. However, the RNG model provided a better prediction of velocities near the front corner above the building, where strong flow separation occurred. Figure 1g–i display the streamlines and normalized turbulent kinetic energy obtained by CFD simulations in the center plane of the domain. Excessive generation of turbulent kinetic energy in the front corner of the building was observed in the standard *k*–*ε* model. Considerable overestimations of *k* in the standard *k*–*ε* model have also been reported by Mochida et al. [34] and Tominaga et al. [35] in their simulations of wind flow around a high-rise building. Compared with the standard *k*–*ε* model, the results of the LK and RNG *k*–*ε* models exhibited considerably lower *k* values, and the effective reduction of *k* in the LK *k*–*ε* model was because of the modification in Equation (5).

A clear recirculation was observed near the front corner above the building in the simulation conducted with the RNG *k*–*ε* model; it was also shown in the results of the LK *k*–*ε* model, but it could not be predicted by the standard *k*–*ε* model. Overall, the wind flow predicted by the standard and LK *k*–*ε* models was better than that predicted by the RNG model, especially in the wake region behind the building. Compared with the standard *k*–*ε* model, the LK model could provide a better prediction of turbulent kinetic energy; thus, it has the potential to improve the simulated results. As a result, the LK *k*–*ε* model was selected in this study to investigate the wind flow and particle dispersion around the human body.

## 3. Methodology and Computational Setups

### 3.1. Transport Equations of the Discrete Phase

This study treated the particles as inert ones, and evaporation and collisions between particles were not considered. This study used an Euler–Lagrange approach to simulate the wind flow and particle dispersion. The fluid phase was treated as a continuum by solving the Navier–Stokes equations, while the motion equation of the discrete phase was solved by tracking the trajectories of a large number of particles according to Newton’s second law as follows:

(8) $$m_{p}\frac{d{\vec{u}}_{p}}{dt}={\vec{F}}_{D}+{\vec{F}}_{G}+{\vec{F}}_{B}+{\vec{F}}_{S}$$

In which $m_{p}=\pi \rho_{p}d_{p}^{3}/6$ is the mass of a particle, ${\vec{u}}_{p}$ is the velocity of a particle in a vector form and *ρ_p_* and *d_p_* are the density and diameter of a droplet, respectively. The terms on the right side of the equation correspond to all the forces exerted onto a particle by the fluid phase. These forces include the drag force ${\vec{F}}_{D}$ caused by friction, the gravity force ${\vec{F}}_{G}\left(=m_{p}\vec{g}\right)$, the buoyant force ${\vec{F}}_{B}$ and the additional forces ${\vec{F}}_{S}$. If the droplets are considered to be spheres and noting that the direction of the drag force is always opposite to the direction of the relative velocity, then the spherical drag law is used to obtain the drag force as follows:

(9) $${\vec{F}}_{D}=-\frac{1}{8}\pi C_{D}\rho d_{p}^{2}\times \left|{\vec{u}}_{p}-\vec{u}\right|\left({\vec{u}}_{p}-\vec{u}\right)$$

In which *C_D_* is the drag coefficient, *ρ* is the density of air and $\vec{u}$ is the vector velocity of the fluid phase. The buoyant force is calculated according to Archimedes’s law as follows:

(10) $${\vec{F}}_{B}=-\frac{\rho }{\rho_{P}}m_{p}\vec{g}$$

The additional forces exerted onto the particles include virtual mass force, pressure gradient force, Brownian force and Saffman lift force. These additional forces were ignored in this study because the virtual mass and pressure gradient forces are not important when the density of the fluid is much lower than the density of the particles, and the Brownian and Saffman’s lift forces are only important for sub-micron particles.

### 3.2. Simulation Arrangements and Mesh Systems

This study aimed to investigate the synthetic effect of wind speed and social distancing on the infection risk in different styles of a susceptible person. A total of 15 simulations were performed, as shown in Table 1. Cases 1–3 were conducted for an isolated human model to examine the wind speed and the aerodynamic effect of the airflow around a human body on the transport characteristics of the exhaled particles. Cases 4–15 were performed for two human models with different wind speeds and social distancing levels to evaluate the infection risk for different styles of a susceptible person who stood downstream of an infected man. Three inflow wind velocities of *V*_in_ = 1.8, 3.6 and 5.4 m/s were examined in this study. The above-mentioned wind speeds covered a wind level from a light to a moderate breeze, and in these meteorological conditions, the communications between people usually occurred in an outdoor environment. The social distancing levels of *L_d_* = 1 and 2 m were studied because the distances within the range of 2 m were usually suggested by different organizations as the smallest safe distance to prevent the transmission of SARS-CoV-2 between individuals during the COVID-19 pandemic. Although longer social distancing is recommended, longer social distancing was not considered in this study because people usually tend to communicate with each other at a moderate distance to facilitate communication, even during the COVID-19 epidemic.

Because the airflow around a human body may influence particle dispersion, the use of a near-real manikin model and accurate representation of the wind flow around the body is important for the study of droplet transport in outdoor spaces. In order to capture the detailed features of real people and to calculate the heat transmission between the body and indoor environment accurately, some famous virtual manikin models have been designed by the ITO Laboratory [36]. The virtual manikins used in this study were adult male and adult female models in a standing posture, and they were made according to the main parameters designed by the ITO Laboratory. Table 2 compares the models created in this study and that designed by the ITO Laboratory in detail. Except for the detailed posture and surface curves, the manikin models created in this study were quite similar to that designed by the ITO Laboratory, which represented the typical characteristics of East Asian people. The heights of the body *H* are 1.74 and 1.60 m for a male model and a female model, respectively. A ponytail hairstyle was designed for the female model, which made her head width a little wider. According to the experiments conducted by Gupta et al. [6], the mouth opening area during coughing was about 4.00 ± 0.95 cm^2^ for an adult male. A mouth opening area of 6.2 cm^2^, which was a little larger than the tested size in the experiment, was used for the infected man in this study to represent a coughing activity.

**Figure 2:**
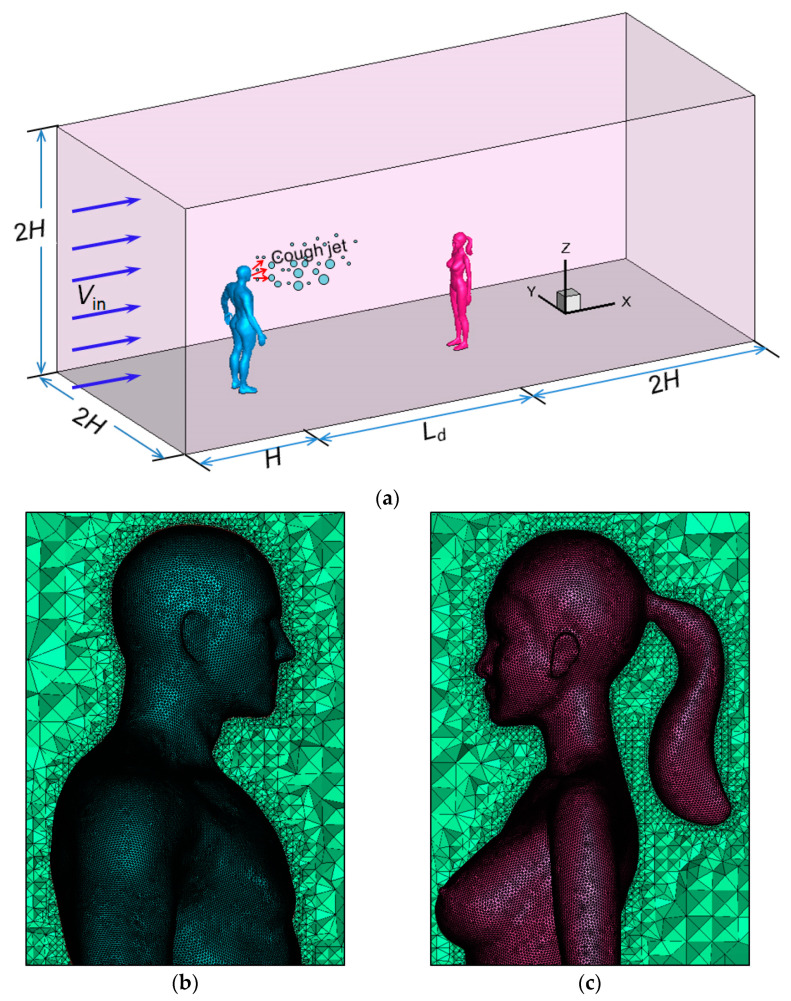
Computational domain and mesh distributions around the human body: (**a**) Computational domain; (**b**) Mesh distributions around the infected man; (**c**) Mesh distributions around a susceptible person.

The domain size and arrangement of the simulation are shown in Figure 2a. An infected man stood in a windy environment with his back facing the oncoming wind, which can cause the riskiest situation to a susceptible person standing downstream [37]. The cough suddenly occurred during communication. This study simulated the transport characteristics of the exhaled droplets using an isolated human model and evaluated the infection risk for a susceptible person standing downstream using two human models. The dimensions of the domain are 2*H* in both the span-wise and vertical directions. The distance between the infected man and the inlet boundary is *H*. For simulations with only an isolated human model, a long domain of 10*H* from the infected man to the outlet boundary was adopted to investigate the dispersion and deposition patterns of the particles. For simulations in which two human models were included, except for the social distancing level *L_d_* between two humans, another distance of 2*H* was used from the susceptible person to the outlet to exclude the influence of the outlet boundary on the flow and dispersion. A short domain downstream of a susceptible person was used because the particles that have left a susceptible person were not of any importance. The zero-coordinate position was set near the heel of the infected man.

Figure 2b,c display the mesh distributions on the surfaces of and around the infected man and a susceptible person downstream. Unstructured mesh systems were used for all simulations. A total number of 5,000,000 and 7,000,000 cells were adopted for Cases 1–3 (isolated human model) and Cases 4–15 (two human models), respectively. For the body surfaces and regions near the human model, denser meshes were adopted to capture the curved geometry configurations and airflow around the manikin. The nondimensional distance *y*^+^ on the body surfaces was between 10 and 30 in most regions for all simulations, and a logarithmic law was used to deal with the near-wall flow. A scalable wall function was adopted to avoid deterioration of the standard wall functions under grid refinement below *y*^+^ < 12.

### 3.3. Boundary Conditions and Numerical Algorithms

Inflow wind velocities of *V*_in_ = 1.8, 3.6, and 5.4 m/s were set to investigate the effect of wind speed on particle dispersion. The top and two lateral boundaries of the domain were set to be symmetrical (the normal gradient was zero for all flow variables). The pressure outlet condition (gauge pressure was zero) was given to the outlet boundary. The envelopes of the human body and the ground were set to the wall, and a wall function was used to deal with the near-wall flow.

**Figure 3:**
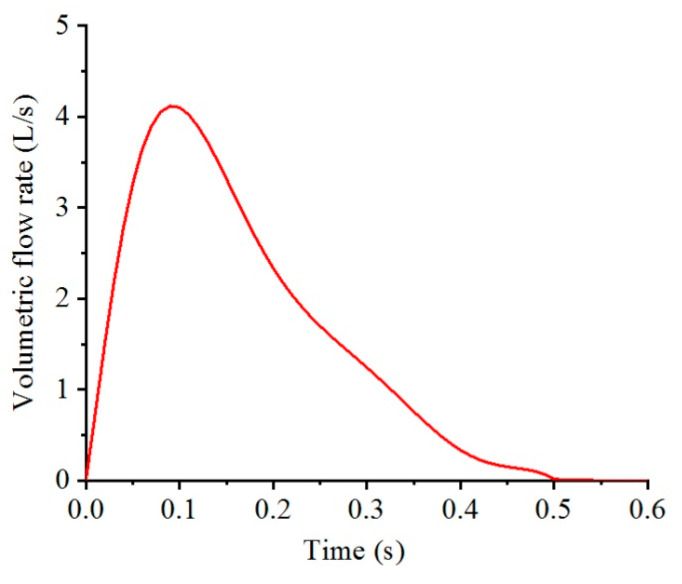
Transient volumetric flow rate of a single cough [6].

A cough activity was selected as the particle source in this study because a cough is one of the prime sources of airborne diseases and produces a large quantity of droplets. The characteristics of a cough-jet are determined by its airflow rate and the number of exhaled droplets in different sizes. Figure 3 presents the cough-jet boundary conditions used in this study at the mouth of the infected man. A transient volumetric flow rate was set for a single cough according to the coughing experiments conducted by Gupta et al. [6]. The number of exhaled droplets of different sizes was determined according to the experiments performed by Duguid [2], which were reproduced by Bourouiba et al. [38]. Detailed particle size and number distributions from one cough are summarized in Table 3. A total of 4973 droplets were exhaled from one cough, and the size of the droplets ranged from several micrometers to thousands of micrometers. The majority of droplets were concentrated at diameters of 8, 16, and 24 μm. The droplets were injected into the flow field at the initial stage of a cough-jet, and the motions of the droplets were determined by the airflow and their weights and buoyancies. A “trap” condition was used for the particles for the ground and envelopes of the human body; while an “escape” condition was adopted for the outlet boundary, which meant that the tracking of a particle was stopped when it reached a wall and an outlet boundary.

The second-order Upwind scheme for the convection term, which is commonly used in wind engineering, was adopted for simulations. The SIMPLE algorithm was used for the pressure–velocity calculations. Steady simulations were first performed to achieve a steady flow field, and they were used as the initial conditions for simulations of particle dispersion. Transient simulations were opened up when coughing started to track the motions of the particles at each time. A time step of Δ*t* = 10^−3^ s was adopted, and 15 iterations were performed in each time step to make the simulations stable. The simulations were stopped when the total time reached 15 s or when there were no particles in the domain.

## 4. Results and Discussion

### 4.1. Airflow and Particle Dispersions around an Isolated Human Model

**Figure 4:**
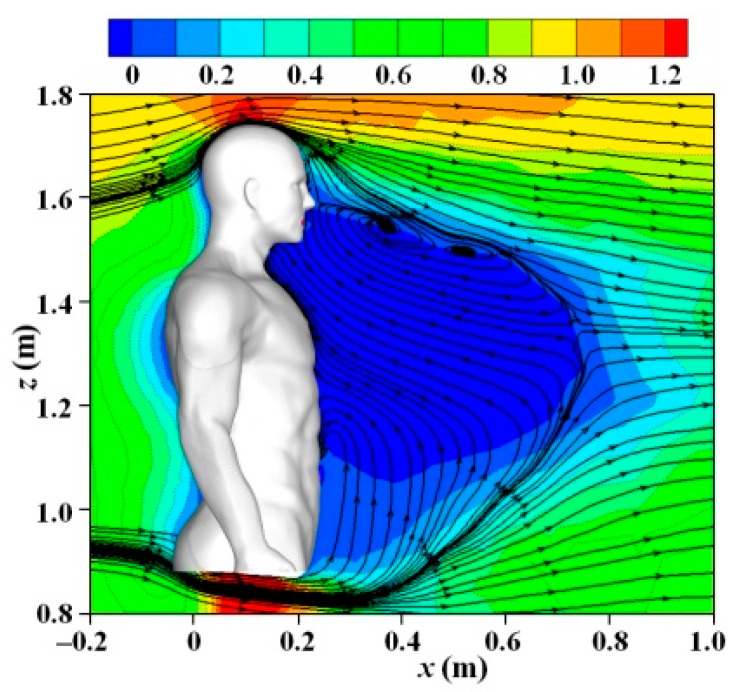
Streamlines and *U*/*V*_in_ in center plane (*y* = 0) around an isolated human model (*V*_in_ = 1.8 m/s).

Figure 4 displays the mean streamlines and *U*/*V*_in_ in the center plane of the domain (*y* = 0) for the case of *V*_in_ = 1.8 m/s. A large area of the reverse-flow region was observed in front of the body, mainly located between the height of the waist and nose, which was probably caused by the upward flow after passing through the two legs. This reverse-flow region lasted around 0.5 m in the streamwise direction, and the entire mouth was immersed in the reverse-flow region, which could strongly affect the dispersion of exhaled particles from the mouth. The separation line between the downward and upward flows was located around the height of the nose position. Strong acceleration of the wind speed was observed between the two legs because of the Venturi effect. The other regions in which the wind velocity was accelerated included the area above and on the two sides of the head.

**Figure 5:**
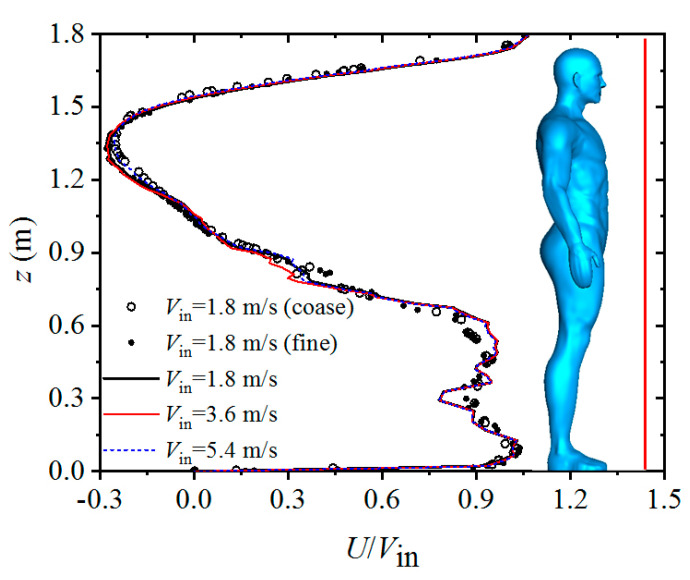
Normalized streamwise velocity in a vertical line in the wake.

Figure 5 presents the normalized streamwise velocity in a vertical line in the wake (about 0.2 m in front of the infected man) for simulations of an isolated human model. To examine the sensitivity of the mesh density, two additional calculations were conducted using a lower number of grids (2,200,000) and a higher number of grids (8,600,000) for the case of *V*_in_ = 1.8 m/s. The results indicated that the calculated flow fields were insensitive to mesh density. For the cases using different inflow wind speeds, the calculated *U*/*V*_in_ in front of the infected man coincided quite well with each other, which indicated that the Reynolds number independent phenomenon was well achieved for the studied range of inflow wind speed. As a result, the reverse flow became stronger in the wake region when the oncoming wind was strong.

**Figure 6:**
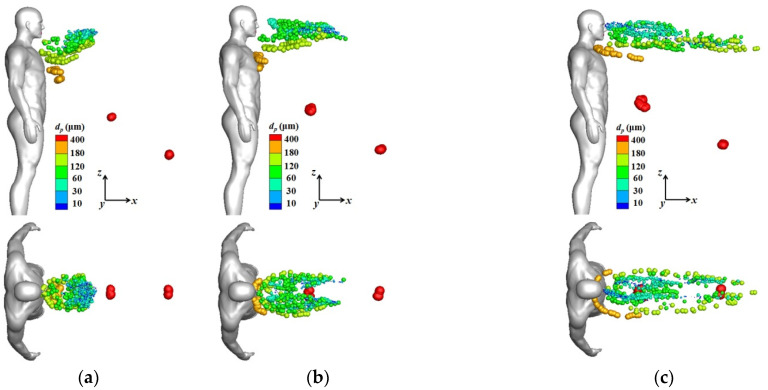

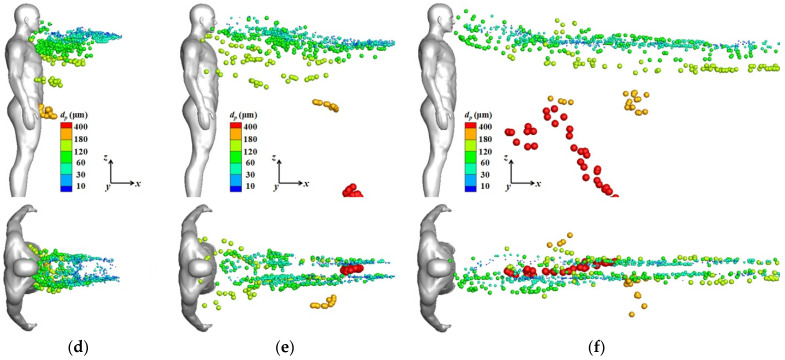
Droplet distributions around an isolated human model (side and top views): (**a**) *V*_in_ = 1.8 m/s, *t* = 0.5 s; (**b**) *V*_in_ = 3.6 m/s, *t* = 0.5 s; (**c**) *V*_in_ = 5.4 m/s, *t* = 0.5 s; (**d**) *V*_in_ = 1.8 m/s, *t* = 1.0 s; (**e**) *V*_in_ = 3.6 m/s, *t* = 1.0 s; (**f**) *V*_in_ = 5.4 m/s, *t* = 1.0 s.

Figure 6 displays the droplet distributions around the isolated human model at times of 0.5 and 1.0 s for the cases of different wind speeds. The particles moved and followed the main wind direction in a narrow zone downstream from the infected man. The dispersion characteristics were quite different for different sizes of particles. Particles larger than 400 μm in diameter were not easily affected by the flow, and they dropped quickly because of their large gravities. Medium particles were more likely affected by both the flow field and their gravities. However, small particles moved and followed the streams because of their small inertia. The particles dispersed widely with the passage of time, and the wind speed had a considerable influence on particle dispersion. At the same instant, the particles were dispersed widely and were blown downstream quickly when the inflow wind was strong. As discussed previously, the reverse and upward flow became stronger in the wake when the inflow wind velocity was larger. As a result, for conditions with a strong wind speed, some medium particles first dropped to the reverse-flow region due to their relatively large gravities and then they were blown back to the body because of the strong reverse flow. As shown in Figure 6a–c, most of the dark-yellow particles were located below the chest and slightly away from the body for Case 1; while they were located near the chest for Case 2 and near the neck for Case 3. The stronger reverse and upward flow in the wake also made some medium particles move to a higher position, which could be clearly observed by the motions of medium particles with green and light-yellow colors. For Cases 2 and 3, many medium particles were even thrown up to a position higher than the eyes by the strong upward flow in the initial stage of coughing because of their large inertia, which could pierce through the streamlines. With the passage of time, the positions of medium particles also began to descend. It was noted that the droplets moved faster on the two sides of the wake in the initial stage because the velocity on the two sides was higher than that in the center location. Although not shown, the results from later time steps showed that most small particles whose diameters were less than 60 μm could be transported downstream at a very long distance, and there was still no deposition tendency for the very small particles less than 10 μm. As a result, no safety distance can be assured for a susceptible person who stands downstream during communication. Except that some medium particles were found to be transported above the eyes in the initial stage under strong wind conditions, as shown in Figure 6, all particles were moving at a level below the mouth height in downstream stations. As a result, a person can avoid being infected by the virus-containing droplets through airborne transmission if they have a greater height. The results also indicated that a strong wind speed can carry larger droplets far away and increase the exposure risk for people standing downstream.

**Figure 7:**
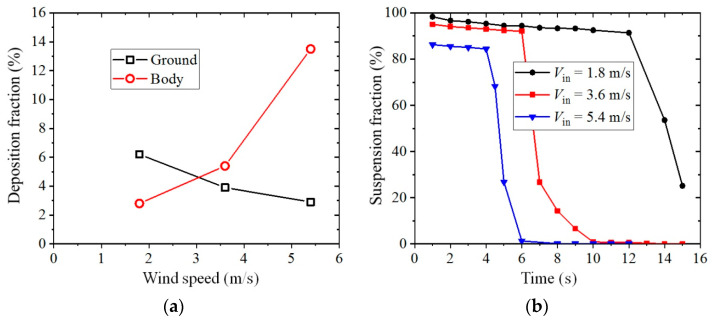
Deposition and suspension fractions (the isolated human model): (**a**) Deposition fraction on the body and ground; (**b**) Suspension fraction with time duration.

Figure 7a presents the deposition fractions on the human body and ground. It is clear that the number of droplets deposited on the ground gradually decreased as the inflow wind velocity increased because more medium particles have the chance to be blown downstream and escape from the domain under strong wind conditions. The number of particles deposited on the human body gradually increased when the inflow wind velocity increased from 1.8 to 3.6 m/s, and it suddenly increased to a high fraction of about 14% for the case of *V*_in_ = 5.4 m/s. The increase in the number of depositions on the human body was mainly caused by the strengthening of the reverse and upward flow in the wake under strong wind conditions. For the strongest wind condition studied in this research (*V*_in_ = 5.4 m/s), depositions of the droplets were even found on the nose of the infected man, which led to the possibility that these particles could be inhaled by the infected man once again. Figure 7b shows the fractions of suspended particles at each time under each wind condition. An initial gradual decrease and then a sudden decrease at some instant for suspension fraction was observed for all simulations. The gradual decrease in the suspension fraction during the first stage was mainly because of depositions of some large and medium particles on the ground. However, the sudden decrease in the number of particles in the domain occurred because many small and medium particles had been blown to the outlet boundary, and they escaped from the domain in groups. The time at which the suspension fraction suddenly decreased was about 12, 6 and 4 s for the cases of *V*_in_ = 1.8, 3.6 and 5.4 m/s, respectively. As a result, a strong wind can blow the exhaled droplets downstream more quickly.

### 4.2. Droplet Transmission between the Infected Man and a Susceptible Person

**Figure 8:**
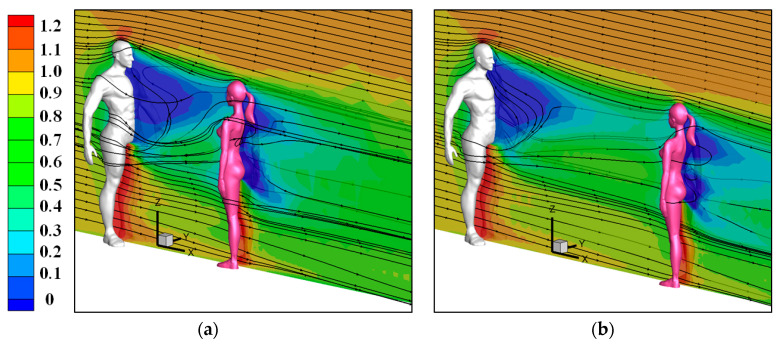
Wind flow around two human models (Male to female model, *V*_in_ = 3.6 m/s): (**a**) 1-m social distancing apart; (**b**) 2-m social distancing apart.

Figure 8 displays the wind flow around the two human models for the cases of *V*_in_ = 3.6 m/s. The wind was accelerated between the two legs of both the upstream and downstream persons because of the Venturi effect. In addition, a large area of the reverse-flow region was observed in front of the infected man in the wake region. From the observations of these three-dimensional streamlines, it is very clear that this reverse-flow area was caused by both the flow coming from regions between the two legs and the arms and human body. Downward flow was observed after flow separation from the top head of the infected man, which made dispersion of the small particles difficult above the head after being exhaled from the mouth. Susceptible persons standing downstream were totally located in the wake of the infected man. A reverse-flow region having a hump shape was observed behind the female model that stood downstream mainly because of the small waist-hip ratio in a female model. For the case with 1-m social distancing, the reverse-flow region in front of the infected man was interrupted a bit by the downstream person.

**Figure 9:**
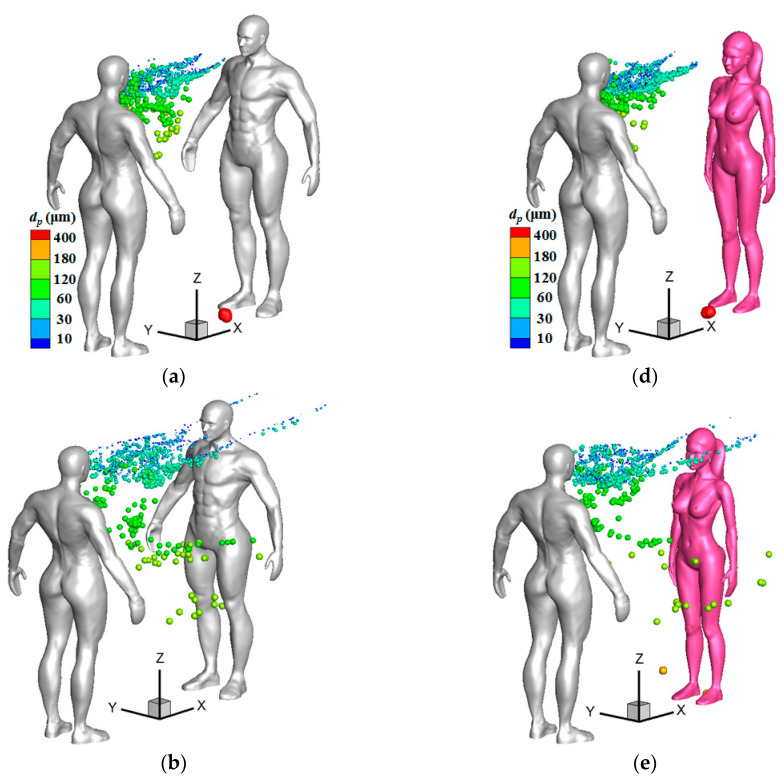

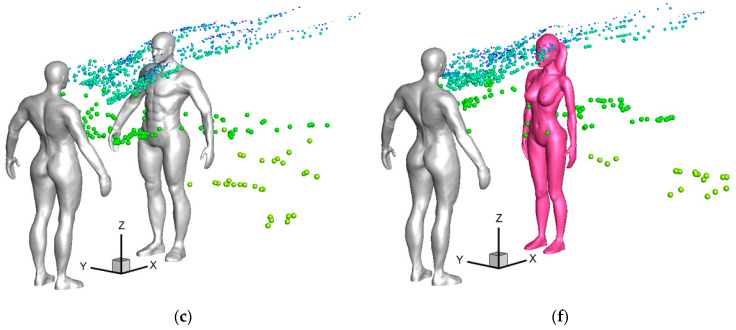
Droplet distributions around two persons at different times (*L_d_* = 1 m, *V*_in_ = 1.8 m/s): (**a**) Male to male model, *t* = 1.0 s; (**b**) Male to male model, *t* = 2.0 s; (**c**) Male to male model, *t* = 3.0 s; (**d**) Male to female model, *t* = 1.0 s; (**e**) Male to female model, *t* = 2.0 s; (**f**) Male to female model, *t* = 3.0 s.

**Figure 10:**
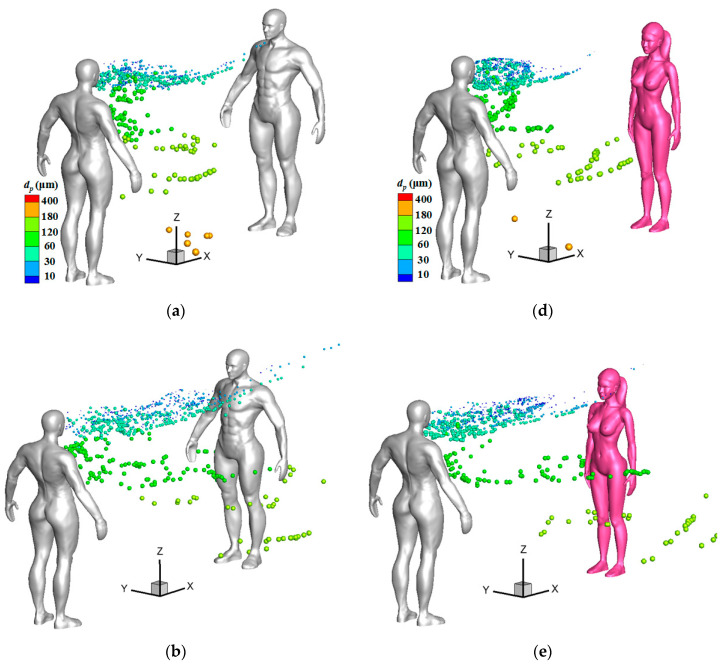

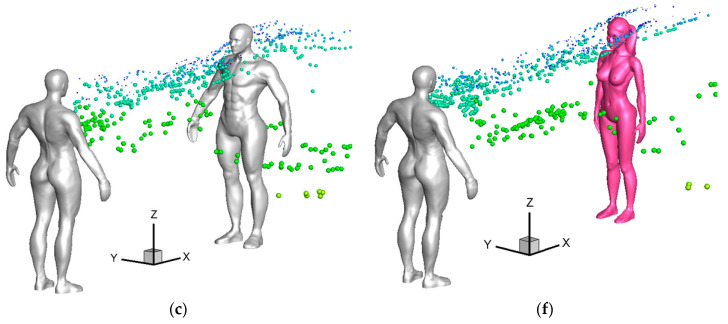
Droplet distributions around two persons at different times (*L_d_* = 2 m, *V*_in_ = 1.8 m/s): (**a**) Male to male model, *t* = 2.0 s; (**b**) Male to male model, *t* = 3.0 s; (**c**) Male to male model, *t* = 4.0 s; (**d**) Male to female model, *t* = 2.0 s; (**e**) Male to female model, *t* = 3.0 s; (**f**) Male to female model, *t* = 4.0 s.

Figure 9 and Figure 10 display droplet distributions around the two human models at different times when the inflow wind was weak (*V*_in_ = 1.8 m/s) for the cases with 1-m and 2-m social distancing, respectively. The particles gradually dispersed downstream with the passage of time, and the dispersion patterns were obviously different for the particles that were smaller than or larger than 60 μm. Small particles that were less than 60 μm were moving over the chest, and they were more threatening to a susceptible person who stood downstream. Medium particles larger than 60 μm had less chance of arriving at the upper body of a susceptible person under a weak wind condition. It seems that the particles with diameters ranging from 30 to 60 μm could easily be trapped in the regions between the two humans. It was observed that many particles were still suspended in the regions between the two persons at the time of *t* = 4.0 s because of the reverse flow in front of the infected man. The vanguard of small particles began to pass through the upper body from about *t* = 2.0 s for the case with 1-m social distancing and from *t* = 3.0 s for the case with 2-m social distancing. As a result, the arrival time of the virus-containing droplets can be extended when the two persons stand at a larger social distance, which can provide a longer time for a susceptible person to cover his/her mouth and nose. Because the small particles have less inertia and are more easily affected by the wind flow, most small particles were moving along the two sides of the head or neck when they passed through the susceptible person. Small particles were mainly moving below the nose for the case with 1-m social distancing and at the height of the neck for the case with 2-m social distancing when they passed through the male model. However, most small particles passed through the two sides of the face of the female. As a result, the infection risk was greater for a susceptible female or person who has a relatively shorter height because the virus-containing droplets can more easily be inhaled by the mouth and nose that are located on the face.

**Figure 11:**
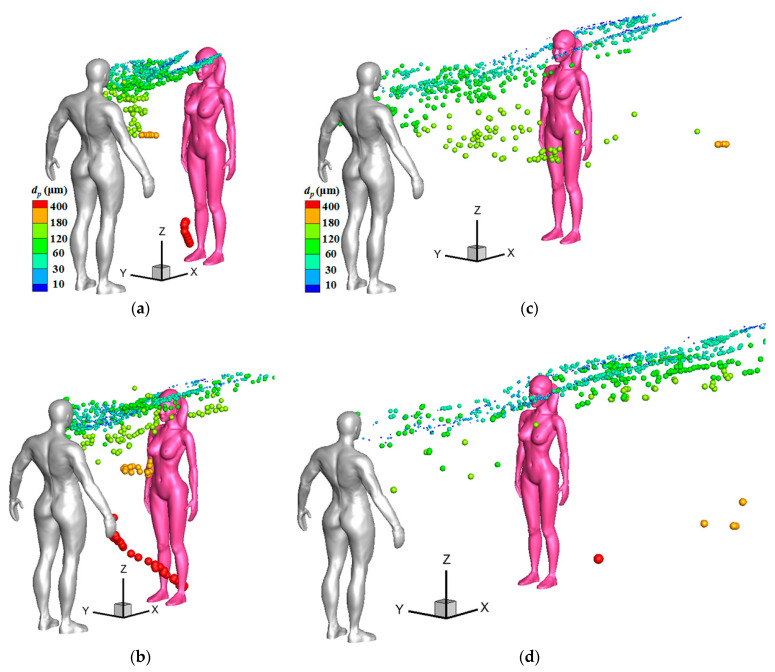
Droplet distributions around a female model under stronger wind conditions: (**a**) *L_d_* = 1 m, *V*_in_ = 3.6 m/s, *t* = 1.0 s; (**b**) *L_d_* = 1 m, *V*_in_ = 5.4 m/s, *t* = 1.0 s; (**c**) *L_d_* = 2 m, *V*_in_ = 3.6 m/s, *t* = 2.0 s; (**d**) *L_d_* = 2 m, *V*_in_ = 5.4 m/s, *t* = 2.0 s.

Figure 11 displays droplet distributions around a female model under stronger wind conditions at some representative instants, at which time many particles passed through a susceptible female. Compared to the situations under a weak wind condition shown in Figure 9d and Figure 10d, it is obvious that many medium particles whose diameter were larger than 60 μm were transported to a higher position in the initial stage and had more chance of being thrown towards the face of a susceptible person under a stronger wind condition. In Figure 11a,c (*V*_in_ = 3.6 m/s), most green particles, the diameters of which ranged from 60 to 120 μm, had passed through the two sides of the face of a susceptible female. In Figure 11b,d (*V*_in_ = 5.4 m/s), most light-yellow particles, the diameters of which ranged from 120 to 180 μm, had passed through the face of a susceptible female. For the cases with 1-m social distancing, some medium particles were also found to move along the two sides of the head when the downstream person was a susceptible male in strong wind conditions.

**Figure 12:**
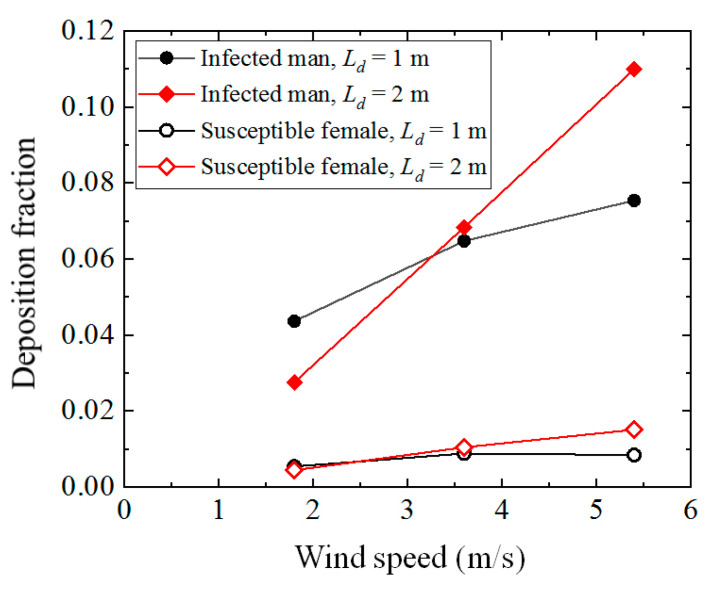
Deposition fractions on the body of an infected man and a susceptible female.

Figure 12 presents the changes in deposition fractions on the body of an infected man and a susceptible female as the wind speed increased. For both the cases with 1-m and 2-m social distancing, the deposition fraction on the infected man increased with the increase in the inflow wind speed, which exhibited a similar tendency to that shown in Figure 7a for cases of an isolated human model. The slight difference between the two figures may be because of flow interruptions by a susceptible person downstream for the cases of two human models. The deposition fraction of a susceptible person downstream also exhibited an increase with the wind speed, but both the number of depositions and the increasing speed of deposition fractions were much smaller than that shown for an infected man. An increase in the deposition fraction on both the infected man and susceptible person may increase the infection risk among other people through contact transmission by touching under a stronger wind condition. It is worth noting that compared with the cases with 1-m social distancing, the deposition fraction increased slightly when the social distancing level was 2 m under a stronger wind speed. Therefore, when evaluating the social distancing effect on the infection risk, the meteorological conditions should be considered in a windy environment. The research conducted by Feng et al. [29] showed that the deposition fractions on both the infected man upstream and a susceptible male downstream decreased considerably when the wind speed increased from 3.9 to 5.5 m/s if evaporation and condensation were considered. As a result, more research is still needed to understand the evaporation and condensation effects on the fates of exhaled particles.

**Figure 13:**
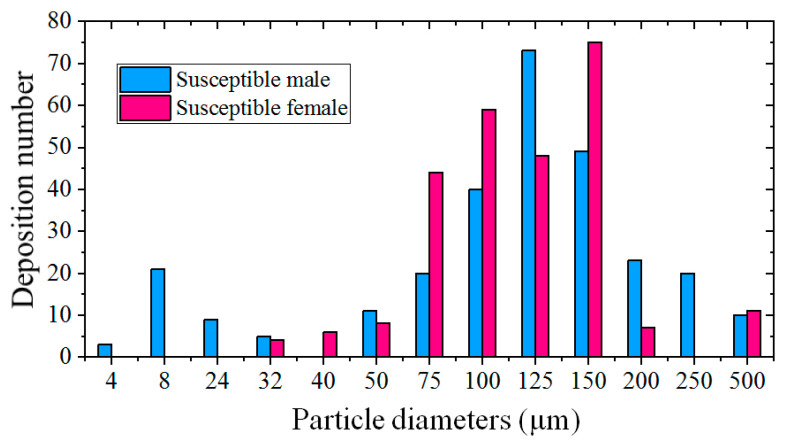
Total number of different sizes of particles deposited on a susceptible person.

The deposition numbers of different sizes of droplets on a susceptible person are presented in Figure 13 to study the infection risk of each kind of droplet. Each bar in Figure 13 was a summation of the number from the total of six simulations for each sex of a susceptible person. It was observed that the number of depositions on a susceptible person was lower for particles less than 60 μm because small particles had small inertia, and they usually moved towards the two sides of the body due to flow separation. The number of depositions was also lower for particles larger than 400 μm because these large particles dropped quickly because of their large gravities. The depositions of the particles with a 500 μm diameter were only found under a strong wind condition and with short social distancing, and the deposited positions were only on the legs. Most of the depositions occurred under stronger wind conditions of *V*_in_ = 3.6 and 5.4 m/s. The droplets with diameters ranging from 75 to 150 μm were more likely to be deposited on the body of a susceptible person because these medium droplets can be easily affected by both the wind flow and their inertia, and they have more chance of piercing through the streamlines.

The number of particles deposited on the face of a susceptible person is listed in Table 4 to judge the extremely risky situations. For the six simulations of male to male models, the depositions on the face of the susceptible male were found in only one case (*L_d_* = 1 m, *V*_in_ = 5.4 m/s). However, particle depositions were found on the face of the susceptible female in most of the cases. The diameters of the deposited particles on the face of the susceptible female ranged from 32 to 150 μm, and most particles were larger than 60 μm. The deposition of medium particles larger than 60 μm occurred mainly because of the strong wind, which first transported these medium particles to a higher position and then threw them directly on the face of the susceptible female due to the relatively shorter height of the female model. As a result, the infection risk is somewhat greater when the downstream person is a susceptible female. Compared to the cases with 1-m social distancing, the 2-m social distancing appeared to reduce the particle (especially for particles with a relatively large diameter) depositions on the face.

### 4.3. Exposure Fractions

Except for direct depositions on the body and face of a susceptible person, another important index called exposure fraction was also used to evaluate the infection risk from airborne transmission. The calculation of the exposure fraction is as follows:

(11) $$f_{e,t}=\frac{n_{t}}{N}$$

where *f*_*e,t*_ is the exposure fraction, and *n_t_* is the total number of particles that are suspended in the breathing zone of a susceptible person at an instant. *N* = 4973 was the total number of particles that were exhaled by one cough. A distance between the spatial locations of a particle and the nose less than 0.2 m was used to judge whether a particle was located inside the breathing zone or not. The particles suspended in the breathing zone can be more threatening to a susceptible person because these particles have more chance of being inhaled by a person or of being deposited on the face of a person if he or she moves slightly.

**Figure 14:**
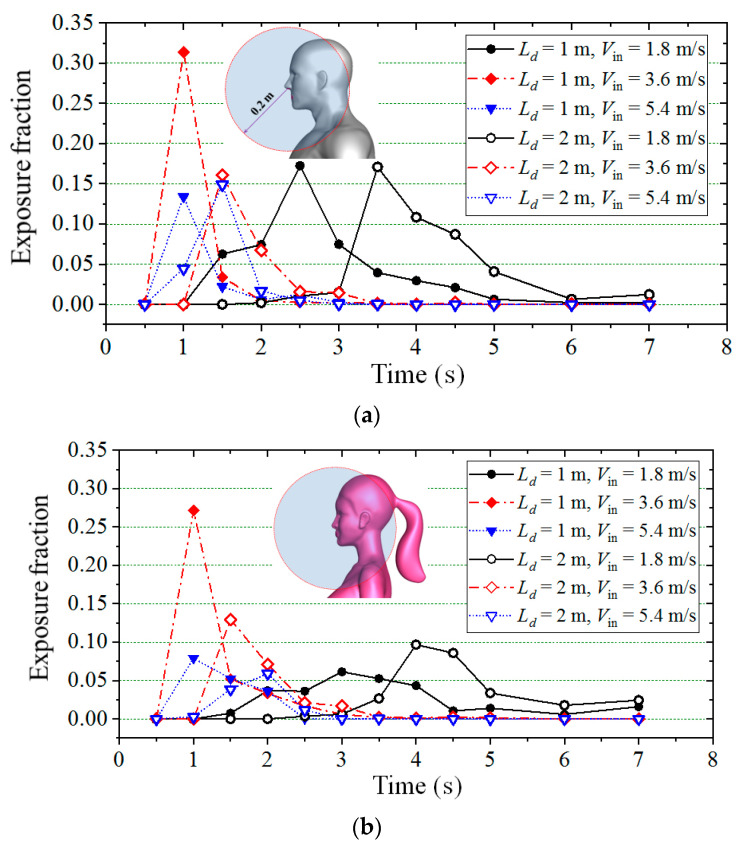
Exposure fractions for a susceptible person at different times: (**a**) Male to male model; (**b**) Male to female model.

Figure 14a,b present the exposure fractions for a susceptible male and susceptible female at different instants, respectively, in which the solid symbols indicate the cases with 1-m social distancing and the hollow symbols indicate the cases with 2-m social distancing. Black, red and blue colors show the cases for *V*_in_ = 1.8, 3.6 and 5.4 m/s, respectively. Overall, similar distribution patterns of the exposure fraction were observed for both male and female models. For all simulations, the exposure fraction had a peak value at a time when many small and some medium particles passed through the head in groups, and the exposure risk was highest for a susceptible person who stood downstream. The highest peak value appeared for the case with 1-meter social distancing and 3.6 m/s inflow wind speed. For most of the meteorological conditions, the effect of social distancing on the peak value of the exposure fraction was small except when the inflow wind speed was equal to 3.6 m/s, in which situations the peak value dropped when the social distancing level extended from 1 to 2 m. It is worth noting that compared with the situation of 1-m social distancing, a considerable increase in the peak value was observed for the case of *V*_in_ = 1.8 m/s when the downstream person was a female and a slight increase in the peak value was observed for the case of *V*_in_ = 5.4 m/s when the downstream person was a male when the social distancing level was 2 m. Although the social distancing effect on the infection risk is not clear, longer social distances can obviously delay the arrival time for the peak value of the exposure fraction under the same wind condition. It was noticed that for the cases with the same social distancing, although the peak value occurred later, the waves were wider when the inflow wind was weak, which means that a longer risky time will be provided under a weak wind condition. The exposure fractions decreased quickly after the time *t* = 6 s, and they were only slightly greater than zero for all simulations. For the cases with the same social distancing and under the same wind condition, the values of exposure fraction were slightly greater when the susceptible person was a male.

**Figure 15:**
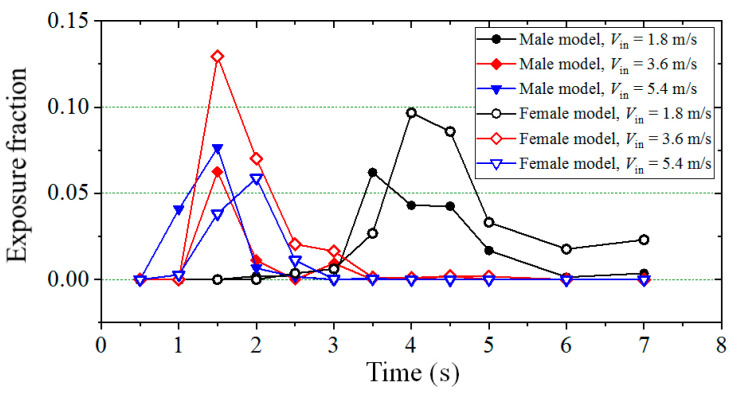
Exposure fractions contributed by particles above the neck (*L_d_* = 2 m).

For the cases with 2-m social distancing, as shown in Figure 10, most small particles were moving at the height of the neck and face positions while passing through a susceptible male and female, respectively. As a result, compared with the situation of a female model, a higher value of the exposure fraction for a male does not absolutely mean a higher infection risk. The exposure fractions contributed by the particles in the breathing zone but above the neck are presented in Figure 15 for the cases with 2-m social distancing, in which the solid symbols were the results of male models and the hollow symbols were the results of female models. Black, red and blue colors indicate the wind conditions of *V*_in_ = 1.8, 3.6 and 5.4 m/s, respectively. If we use the bottom height of the jaw as the separation line, the particles located in the breathing zone but above this height can generate more infection risk in a susceptible person. As shown in Figure 15, except under the condition of *V*_in_ = 5.4 m/s, in which the difference between the exposure fraction of a male and female was small, the exposure fraction contributed by the particles above the neck was obviously larger when the downstream person was a female. As a result, for the cases with larger social distancing, the infection risk was usually greater when a female stood downstream of an infected man in most of the situations. It should be stated that simulations with 2-m social distancing under a weak wind condition in this study had more realistic significance because people usually communicate with each other under the condition of a lower outdoor wind speed and with moderate social distancing.

**Figure 16:**
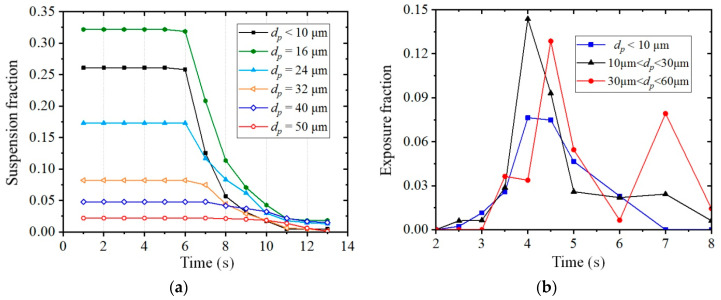
Suspension fraction and exposure fraction of small particles at a time duration (male to female model, *L_d_* = 2 m, *V*_in_ = 1.8 m/s): (**a**) Suspension fraction of small particles; (**b**) Exposure fractions from different sizes of small particles.

As small particles are more likely to be breathed in through the mouth or nose and contribute the most to airborne transmission of viruses, the dispersion characteristics of small particles with a diameter of less than 60 μm were analyzed in detail for the male to female model case with 2-m social distancing under the wind condition of *V*_in_ = 1.8 m/s, as shown in Figure 16. Figure 16a presents the suspension fraction of different small particles with the time duration. Small particles whose diameters were less than 30 μm exhibited similar dispersion patterns, and their suspension fractions dropped suddenly from the time *t* = 6.0 s. As the diameter of the particle increased, the time at which the suspension fraction began to decrease was delayed, and the decreasing speed of the suspension fraction became lower. It was worth noting that for particles with diameters of 40 and 50 μm, although their total fractions were small, they could stay or were trapped for a longer time between two persons, and as a result, they could be constantly threatening to a susceptible person downstream. The time at which the suspension fraction began to decrease was even delayed to *t* = 10 s for the particles with a diameter of 50 μm.

The exposure fractions from different ranges of particles are presented in Figure 16b, in which the exposure fractions were obtained by the number of particles located in the breathing zone, divided by their individual numbers but not the total number *N*. It was observed that the exposure fractions of particles whose diameters were less than 10 μm and ranged from 10 to 30 μm had a peak value at the time around *t* = 4.0 s, but the peak value was slightly delayed and another peak value was observed at the time around *t* = 7.0 s for the particles ranging from 30 to 60 μm. The peak values of exposure fractions were about 0.076, 0.144 and 0.13 for the particles that were less than 10 μm, ranged from 10 to 30 μm and from 30 to 60 μm, respectively. This means that compared with the extremely small particles (*d_p_* < 10 μm), the relatively larger particles are more likely to approach the breathing zone when passing through the body and can be more threatening to a susceptible person. Two peak values of the exposure fraction for the particles ranging from 30 to 60 μm indicated a long duration of exposure risk generated by these particles.

## 5. The Limitations

Although various cases were conducted, this study still has limitations. The turbulent flow around a blunt body cannot be accurately captured by a RANS turbulence model. As a result, large-eddy simulation is necessary to be conducted in future studies. For real situations, airflow and particle dispersions may be affected by other factors, such as the body features, the human posture, the initial injection method for the particles and the braking effect on local airflow by a surgical mask. Further studies on the influence of these factors can help to thoroughly understand the transmission characteristics of respiratory droplets in outdoor environments. This study only concerned the dispersion of particles generated from one cough. Particle dispersion caused by a continuous source, such as talking and breathing, is also necessary to be investigated. An additional limitation of this study is that the evaporation effect was not considered, while the evaporation effect becomes important for an environment with lower humidity.

## 6. Conclusions

This study used transient CFD simulations to investigate the impact of wind speed and airflow around a human body on the dispersion of coughed droplets in an outdoor space. The influences of social distancing on the infection risk in different susceptible persons were evaluated under different wind conditions based on the direct deposition on the body and the exposure fraction around the nose. The exposure risks from different sizes of particles were also discussed. The main conclusions of this study are as follows:

(1) A reverse and upward flow was created in the wake of the infected person, and it became stronger as the wind speed increased. The enhanced reverse and upward flow transported more particles backward and more medium particles to a higher position and increased the number of depositions on a human body. Stronger wind can blow the particles downstream quickly and reduce the number of particles deposited on the ground.
(2) The dispersion patterns of particles with different sizes were different. Medium particles larger than 60 μm had less chance of reaching the upper body of a susceptible person under a weak wind condition, while many of them can be transported to higher locations and have more chance of being thrown toward the face of a susceptible person under a stronger wind condition. Particles smaller than 60 μm were moving at the height of the head or neck as they passed through a susceptible person. Particles with diameters ranging from 30 to 60 μm were trapped for a longer time between two persons and were more likely to approach the breathing zone of a susceptible person; as a result, they can be largely and continuously threatening. Many small and some medium particles passing through the breathing zone of a susceptible person simultaneously led to the peak value of the exposure fraction.
(3) The deposition of particles on both an infected man and a susceptible person increased with the increase in wind speed. Most of the depositions occurred under stronger wind conditions. The particle depositions were rarely observed on the face of a susceptible male, but they were found on the face of a susceptible female in most of the cases. Droplets with diameters ranging from 75 to 150 μm were more likely to deposit on the body of a susceptible person.
(4) Compared with the cases with 1-m social distancing, the deposition fraction increased slightly when the wind speed was larger, and an increase in the peak value of the exposure fraction was observed for a susceptible person in some situations when the social distancing was 2 m. Longer social distancing can alleviate the deposition of particles on the face and delay the arrival of the riskiest time for a susceptible person. As a result, the effect of social distancing on the infection risk was not distinct in a windy environment.
(5) The infection risk was evaluated for different styles of a susceptible person in a windy condition. Stronger wind had the potential to create a greater risk of instantaneous exposure. A susceptible person can avoid being infected by the virus-containing droplets through airborne transmission if they have a greater height. Compared with the situations for a susceptible male, the infection risk was somewhat greater for a susceptible female that had a shorter height because more particles were deposited on the face and the exposure fractions contributed by the particles above the neck were greater in most cases.

This study discussed the infection risk of the SARS-CoV-2 virus that was contained in the droplets generated by a cough activity. The results and conclusions of this study can be of valuable reference for the assessment of infection risk to other types of bacteria, such as influenza virus, from airborne transmissions.

## Figures and Tables

**Table 1:** Simulation cases.

		Inflow Wind Speed		
		*V*_in_ = 1.8 (m/s)	*V*_in_ = 3.6 (m/s)	*V*_in_ = 5.4 (m/s)
Isolated human model		Case 1	Case 2	Case 3
Male to male	*L_d_* = 1 m	Case 4	Case 5	Case 6
	*L_d_* = 2 m	Case 7	Case 8	Case 9
Male to female	*L_d_* = 1 m	Case 10	Case 11	Case 12
	*L_d_* = 2 m	Case 13	Case 14	Case 15

**Table 2:** Detailed parameters of the manikin models used in this study.

	Male Model		Female Model	
	This Study	ITO [36]	This Study	ITO [36]
Whole body area (m^2^)	1.98	1.75	1.42	1.32
Body height *H* (m)	1.74	1.74	1.60	1.59
Waist height (m)	1.14	1.13	1.00	1.03
Waist width (m)	0.265	0.268	0.192	0.197
Head width (m)	0.15	0.15	0.16	0.14
Mouse area (m^2^)	0.00062	—	0.00055	—
Mouse width (m)	0.042	0.052	0.035	0.042
Hair	No	No	Yes	No

**Table 3:** Particle size and number distributions from one cough [38].

*d_p_* (μm)	2	4	8	16	24	32	40	50
Number	50	290	970	1600	870	420	240	110
*d_p_* (μm)	75	100	125	150	200	250	500	1000
Number	140	85	48	38	35	29	34	14

**Table 4:** Number of particles deposited on the face of a susceptible person.

		Cases		
		*V*_in_ = 1.8 m/s	*V*_in_ = 3.6 m/s	*V*_in_ = 5.4 m/s
Male to male	*L_d_* = 1 m	0	0	2
	*L_d_* = 2 m	0	0	0
Male to female	*L_d_* = 1 m	4	4	16
	*L_d_* = 2 m	0	0	20

## Data Availability

The CAD model of manikins and raw data requests can be made to the corresponding author.

## References

[1] Doremalen N.V., Bushmaker T., Morris D.H., Van Doremalen N., Bushmaker T., Morris D.H., Holbrook M.G., Gamble A., Williamson B.N., Munster V.J. (2020). Aerosol and surface stability of SARS-CoV-2 as compared with SARS-CoV-1. N. Engl. J. Med..

[2] Duguid J.P. (1964). The size and the duration of air-carriage of respiratory droplets and droplet-nuclei. Epidemiol. Infect..

[3] Yang G.W.M., Lee C.M., Chen C.C., Wu K.P. (2007). The Size and Concentration of Droplets Generated by Coughing in Human Subjects. J. Aerosol Med..

[4] Xie X.J., Li Y.G., Sun H.Q., Liu L. (2009). Exhaled droplets due to talking and coughing. J. R. Soc. Interface.

[5] Chao C.Y.H., Wan M.P., Morawska L., Johnson G.R., Ristovski Z.D., Hargreaves M., Mengersen K., Corbett S., Li Y., Xie X. (2009). Characterization of expiration air jets and droplet size distributions immediately at the mouth opening. J. Aerosol Sci..

[6] Gupta J.K., Lin C.H., Chen Q. (2009). Flow dynamics and characterization of a cough. Indoor Air.

[7] Gupta J.K., Lin C.H., Chen Q. (2010). Characterizing exhaled airflow from breathing and talking. Indoor Air.

[8] Han M., Ooka R., Kikumoto H., Oh W., Bu Y.C., Hu S.Y. (2021). Measurements of exhaled airflow velocity through human coughs using particle image velocimetry. Build. Environ..

[9] Cheng C.H., Chow C.L., Chow W.K. (2020). Trajectories of large respiratory droplets in indoor environment: A simplified approach. Build. Environ..

[10] Liu Z.J., Liu H.Y., Rong R., Cao G.Q. (2020). Effect of a circulating nurse walking on airflow and bacteria-carrying particles in the operating room: An experimental and numerical study. Build. Environ..

[11] Liu Z.J., Wang L.Q., Rong R., Fu S.F., Cao G.Q., Hao C.C. (2020). Full-scale experimental and numerical study of bioaerosol characteristics against cross-infection in a two-bed hospital ward. Build. Environ..

[12] Zhang Z., Chen X., Mazumdar S., Zhang T.F., Chen Q. (2009). Experimental and numerical investigation of airflow and contaminant transport in an airliner cabin mockup. Build. Environ..

[13] Gupta J.K., Lin Q.C., Chen H. (2011). Transport of expiratory droplets in an aircraft cabin. Indoor Air.

[14] Yang L., Li X.D., Yan Y.H., Tu J.Y. (2018). Effects of cough-jet on airflow and contaminant transport in an airliner cabin section. J. Comput. Multiph. Flows.

[15] Yan Y.H., Li X.R., Yang L., Yan P., Tu J.Y. (2020). Evaluation of cough-jet effects on the transport characteristics of respiratory-induced contaminants in airline passengers’ local environments. Build. Environ..

[16] Zhang L., Li Y.G. (2012). Dispersion of coughed droplets in a fully-occupied high-speed rail cabin. Build. Environ..

[17] Yang X., Ou C.Y., Yang H.Y., Liu L., Song T., Kang M., Lin H.L., Hang J. (2020). Transmission of pathogen-laden expiratory droplets in a coach bus. J. Hazard. Mater..

[18] Liu S.M., Zhao X.W., Nichols S.R., Bonilha M.W., Derwinski T., Auxier J.T., Chen Q. (2022). Evaluation of airborne particle exposure for riding elevators. Build. Environ..

[19] Zhu S.W., Kato S., Yang J.H. (2006). Study on transport characteristics of saliva droplets produced by coughing in a calm indoor environment. Build. Environ..

[20] He Q.B., Niu J.L., Gao N.P., Zhu T., Wu J.Z. (2011). CFD study of exhaled droplet transmission between occupants under different ventilation strategies in a typical office room. Build. Environ..

[21] Zhang Y.X., Feng G.H., Bi Y., Cai Y.L., Zhang Z., Cao G.Y. (2019). Distribution of droplet aerosols generated by mouth coughing and nose breathing in an air-conditioned room. Sustain. Cities Soc..

[22] Shang Y.D., Dong J.L., Tian L., He F.J., Tu J.Y. (2022). An improved numerical model for epidemic transmission and infection risks assessment in indoor environment. J. Aerosol Sci..

[23] Bulfone T.C., Malekinejad M., Rutherford G.W., Razani N. (2021). Outdoor Transmission of SARS-CoV-2 and Other Respiratory Viruses: A Systematic Review. J. Infect. Dis..

[24] Makruf A., Ramdhan D.H. (2021). Outdoor Activity: Benefits and Risks to Recreational Runners during the COVID-19 Pandemic. Natl. Public Health J..

[25] Dominski F.H., Brandt R. (2020). Do the benefits of exercise in indoor and outdoor environments during the COVID-19 pandemic outweigh the risks of infection?. Sport Sci. Health.

[26] WHO Advice for the Public: Coronavirus Disease (COVID-19). https://www.who.int/emergencies/diseases/novel-coronavirus-2019/advice-for-public.

[27] Zhao T.M., Cheng C., Liu H.X., Sun C.Y. (2020). Is one- or two-meters social distancing enough for COVID-19? Evidence for reassessing. Public Health.

[28] Lazzerini M., Putoto G. (2020). COVID-19 in Italy: Momentous decisions and many uncertainties. Lancet Glob. Health.

[29] Feng Y., Marchal T., Sperry T., Yi H. (2020). Influence of wind and relative humidity on the social distancing effectiveness to prevent COVID-19 airborne transmission: A numerical study. J. Aerosol Sci..

[30] Li H., Leong F., Xu G., Ge Z., Kang C., Lim K. (2020). Dispersion of evaporating cough droplets in tropical outdoor environment. Phys. Fluids.

[31] Yang X., Yang H., Ou C., Luo Z., Hang J. (2021). Airborne transmission of pathogen-laden expiratory droplets in open outdoor space. Sci. Total Environ..

[32] Kato M., Launder B.E. The Modelling of Turbulent Flow around Stationary and Vibrating Square Cylinders. Proceedings of the 9th Symposium on Turbulence and Shear Flows.

[33] Shirasawa T., Yoshie R., Tanaka H., Kobayashi T., Mochida A., Endo Y. Cross comparison of CFD results of gas diffusion in weak wind region behind a high-rise building. Proceedings of the Fourth International Conference on Advances in Wind and Structures (AWAS’08).

[34] Mochida A., Tominaga Y., Murakami S., Yoshie R., Ishihara T., Ooka R. (2002). Comparison of various k-ε models and DSM applied to flow around a high-rise building -report on AIJ cooperative project for CFD prediction of wind environment. Wind. Struct..

[35] Tominaga Y., Mochida A., Murakami S., Sawaki S. (2008). Comparison of various revised k-ε models and LES applied to flow around a high-rise building model with 1:1:2 shape placed within the surface boundary layer. J. Wind Eng. Ind. Aerodyn..

[36] http://www.arch.t-kougei.ac.jp/ito/vm.html.

[37] Li F.J., Jiang G.Y., Hu T.T. (2022). Coughing Intensity and Wind Direction Effects on the Transmission of Respiratory Droplets: A Computation with Euler–Lagrange Method. Atmosphere.

[38] Bourouiba L., Dehandschoewercker E., Bush J.W.M. (2014). Violent expiratory events: On coughing and sneezing. J. Fluid Mech..

